# *Staphylococcus equorum* plasmid pKS1030-3 encodes auxiliary biofilm formation and trans-acting gene mobilization systems

**DOI:** 10.1038/s41598-023-38274-8

**Published:** 2023-07-10

**Authors:** Sojeong Heo, Seung-Eun Oh, Gawon Lee, Jinwook Lee, Nam-Chul Ha, Che Ok Jeon, Keuncheol Jeong, Jong-Hoon Lee, Do-Won Jeong

**Affiliations:** 1 Department of Food and Nutrition, Dongduk Women’s University, Seoul, 02748 Republic of Korea; 2 Research Institute of Agriculture and Life Sciences, Center for Food and Bioconvergence, Department of Agricultural Biotechnology, CALS, Seoul National University, Seoul, 08826 Republic of Korea; 3 Department of Life Science, Chung-Ang University, Seoul, 06974 Republic of Korea; 4 Department of Food Science and Biotechnology, Kyonggi University, Suwon, 16227 Republic of Korea

**Keywords:** Applied microbiology, Prokaryote

## Abstract

The foodborne bacterium *Staphylococcus equorum* strain KS1030 harbours plasmid pSELNU1, which encodes a lincomycin resistance gene. pSELNU1 undergoes horizontal transfer between bacterial strains, thus spreading antibiotic resistance. However, the genes required for horizontal plasmid transfer are not encoded in pSELNU1. Interestingly, a relaxase gene, a type of gene related to horizontal plasmid transfer, is encoded in another plasmid of *S. equorum* KS1030, pKS1030-3. The complete genome of pKS1030-3 is 13,583 bp long and encodes genes for plasmid replication, biofilm formation (the *ica* operon), and horizontal gene transfer. The replication system of pKS1030-3 possesses the replication protein-encoding gene *repB*, a double-stranded origin of replication, and two single-stranded origins of replication. The *ica* operon, relaxase gene, and a mobilization protein-encoding gene were detected in pKS1030-3 strain-specifically. When expressed in *S. aureus* RN4220, the *ica* operon and relaxase operon of pKS1030-3 conferred biofilm formation ability and horizontal gene transfer ability, respectively. The results of our analyses show that the horizontal transfer of pSELNU1 of *S. equorum* strain KS1030 depends on the relaxase encoded by pKS1030-3, which is therefore trans-acting. Genes encoded in pKS1030-3 contribute to important strain-specific properties of *S. equorum* KS1030. These results could contribute to preventing the horizontal transfer of antibiotic resistance genes in food.

## Introduction

Antibiotic resistance (AR) among microbes has become one of the biggest threats to global human health. In 2013, the Centers for Disease Control and Prevention of the United States published a report that warned about the spread among environments of foodborne pathogenic bacteria with mobile AR genes, including from foods to humans^1^. The World Health Organization has also recognised the threat of AR and published a global priority list of antibiotic-resistant bacteria in 2017^2^.

Bacteria can acquire new genetically determined traits from other microbes in their ecosystem via horizontal gene transfer (HGT)^3,4^. HGT plays an important role in the spread of AR among bacterial taxa. There are three major/canonical mechanisms of HGT: transformation, via extracellular DNA; transduction, via bacteriophage; and conjugation, via mobile genetic elements such as plasmid. Conjugation is the most common mechanism of HGT, and conjugative transfer of plasmids is the most efficient way of horizontally spreading AR genes^5,6^. Conjugation via plasmids involves the enzyme relaxase, which recognises an origin of transfer (*oriT*) on the plasmid and forms a “relaxosome” complex for gene exchange through binding with *oriT*, the type IV coupling protein, and mating channel components such as the type IV secretion system (T4SS)^7^.

In our previous analysis of cultivable bacteria in high-salt fermented seafood, *Staphylococcus equorum* was identified as a predominant bacterial species^8^. During subsequent safety and functional assessments of *S. equorum* isolates to identify safe and efficient candidate starter strains, a lincomycin resistance gene (encoding lincosamide *O*-nucleotidyltransferase, *lnuA*) was amplified from 4 of 126 isolates^9^. The *lnuA* gene was located on small plasmids, named pSELNU1–3, with only two different nucleotide sequences among these three plasmids^10^. Notably, although pSELNU1 is not a conjugative plasmid, pSELNU1 in *S. equorum* strain KS1030 was transferred to other *Staphylococcus* species, *Enterococcus faecalis*, and *Tetragenococcus halophilus in vitro*^10^, and was also transferred to *S. saprophyticus* during soybean fermentation (in situ) and passage through murine intestines (in vivo) under lincomycin pressure^11^. Recent complete genome sequencing revealed that *S. equorum* KS1030 also harbours plasmid pKS1030-3, which encodes elements facilitating gene mobility such as a relaxase^12^.

In the current study, we characterised pKS1030-3 from *S. equorum* KS1030 on the genomic level and illuminate its involvement in plasmid transfer between species involved in food fermentation, which demonstrates the possibility of horizontal AR gene transfer within food matrices. We show that pKS1030-3 can serve as an auxiliary trans-acting factor for horizontal transfer of pSELNU1 (containing the lincomycin resistance gene *lnuA*), and also that pKS1030-3 may be involved in biofilm formation.

## Materials and methods

### Bacterial strains and culture conditions

*Staphylococcus equorum* strain KS1030, originally isolated from a Korean high-salt fermented seafood, was subjected to genomic and experimental analyses^9^. Here, plasmid pKS1030-3 from *S. equorum* KS1030 was used in assessment of gene transferability and biofilm formation. In gene transfer experiments, *S. saprophyticus* KM1053 was used as the recipient strain because it is resistant to tetracycline^11^. *S. aureus* RN4220 was used as an expression host for constructed plasmids. *Staphylococcus* strains were cultured in tryptic soy broth (TSB; Becton, Dickinson and Co., Franklin Lakes, NJ, USA) at 37 °C for 12 h.

*Escherichia coli* DH5α was used as the cloning host and was cultured in Luria–Bertani broth (Becton, Dickinson and Co.) at 37 °C for 12 h.

### Genomic analyses

The complete genome sequence of *S. equorum* strain KS1030 was published previously (GenBank accession: CP068576–CP068580)^12^. In the current study, the nucleotide sequence of plasmid pKS1030-3 (CP068579) from *S. equorum* KS1030 was analysed. CLgenomics™ software v.1.55 (CJ Bioscience, Inc, Seoul, Korea) and the web-hosted BLAST programs of NCBI were used to find genes and gene products with sequence identity. Rapid Annotation using Subsystem Technology (RAST)^13^ was used to determine gene contents based on functional subsystem classifications. MEGA11 software was used for sequence analyses of DNA and proteins^14^. The sequence identities of deduced amino acid were determined by MegAlign module of Lasergene DNAStar^15^. Protein secondary and tertiary structures were predicted using Predict Secondary Structure (PSIPRED)^16^ and AlphaFold^17^.

### Comparative genomics

For comparative genomic analysis within the species *S. equorum*, genome sequence data for strains KS1030 (GenBank accession: CP068576–CP068580), KM1031 (CP013980–CP013983), C2014 (CP013714–CP013719), KS1039 (CP013114), Mu2 (CAJL01000001–CAJL01000030), and UMC-CNS-924 (AVBD01000001–AVBD01000039) were obtained from the NCBI database (http://ncbi.nlm.nih.gov/genomes). Genes were predicted using the RAST server for SEED-based automated annotation^13^. The predicted genes of strains were confirmed using the iPath (v.3) module^18^, and CLgenomics™ v.1.55 software.

### DNA cloning and transformation

Approximately 3.7-kb fragments containing biofilm formation genes (the *ica* operon) were amplified from pKS1030-3 of *S. equorum *KS1030 using primer set, ica-F and ica-R with restriction enzyme site *Xho*I, (Supplementary Table 1) and then digested by *Xho*I. For construction of plasmids containing the relaxase (*rlx*) gene, the fragment containing mobilization relaxosome protein (*mobC*), *rlx*, and a hypothetical protein (HP) genes was amplified from pKS1030-3 with primers Rlx-F and Rlx-R with *Eco*RV and *Xho*I restriction enzyme site, respectively (Supplementary Table 1). Digested fragments were inserted into the same sites of pYJ335^19^, respectively. The resulting plasmids were named pYJ335-ica and pYJ335-rlx. PCR amplifications of the *ica* operon and fragments containing the *rlx* gene were performed using a T3000 thermocycler (Analytik Jena, Jena, Germany) using an Inclone *Taq* polymerase kit (Inclone Biotech, Seongnam, South Korea) according to the manufacturer’s manual. All PCRs were performed using 30 cycles of denaturing at 95 °C for 1 min, annealing at 60 °C for 2 min, and elongation at 72 °C for 1 min. Constructed plasmid DNA was introduced into *E. coli* DH5α by the method of Hanahan and Meselson^20^, and into *S. aureus* RN4220 by electroporation^21^ with a gene pulser (BioRad, Hercules, CA, USA).

### Biofilm formation analysis

An overnight culture of *S. aureus* RN4220 containing pYJ335-ica in TSB was diluted 200-fold with fresh TSB containing 0.5% glucose. Culture (200 μl) was added to each well of a 96-well microtiter plate and incubated for 24 h at 37 °C without shaking. After the supernatant was discarded, the plates were dried, and the cells were stained with 0.1% crystal violet^22^. For quantitative analysis of biofilm production, stained cells were released by adding 150 μl of 50% dimethylsulfoxide, 100 μl aliquots were transferred to a new microtiter plate, and the optical density at 595 nm was measured^23^. The experiment was conducted three times, independently.

### Plasmid transfer experiments

To determine horizontal plasmid transferability, recipient strain *S. saprophyticus* KM1053^11^ was mated with donor strain *S. aureus* RN4220 (carrying pSELNU1 and pYJ335-rlx) using the broth mating method^24^. *S. aureus* RN4220 (pSELNU1) and *S. aureus* RN4220 (pYJ335-rlx) were used as control donor strains. Donor strains containing pSELNU1 were lincomycin-resistant, and the recipient strain was lincomycin-sensitive and tetracycline-resistant. Therefore, transfer of pSELNU1 confers resistance to lincomycin, which facilitates transconjugant selection. Donor cells in the logarithmic growth phase in Mueller–Hinton (MH) broth (Becton, Dickinson and Co.) were mixed with recipient cells in the logarithmic growth phase (also in MH broth) at a 1:10 ratio and incubated at 30 °C for 3 h. The mixture was spread onto the surface of TSA plates supplemented with 30 mg/l lincomycin and 10 mg/l tetracycline. Transconjugants were selected after incubation at 30 °C for 24 h, and were confirmed by colony PCR with primers for amplification of *lnuA*^9,25^. Recipient traits of transconjugants were confirmed *S. saprophyticus*, not *S. equorum* by 16S rRNA gene sequence analysis.

### Statistical analysis

Duncan’s multiple range test following one-way analysis of variance was applied to evaluate significant differences between the average values obtained in biofilm formation analyses. All statistical analysis was performed using the SPSS software package (v.27.0; IBM SPSS Statistics, Armonk, NY, USA).

## Results and discussion

### Overall features of pKS1030-3

**Figure 1:**
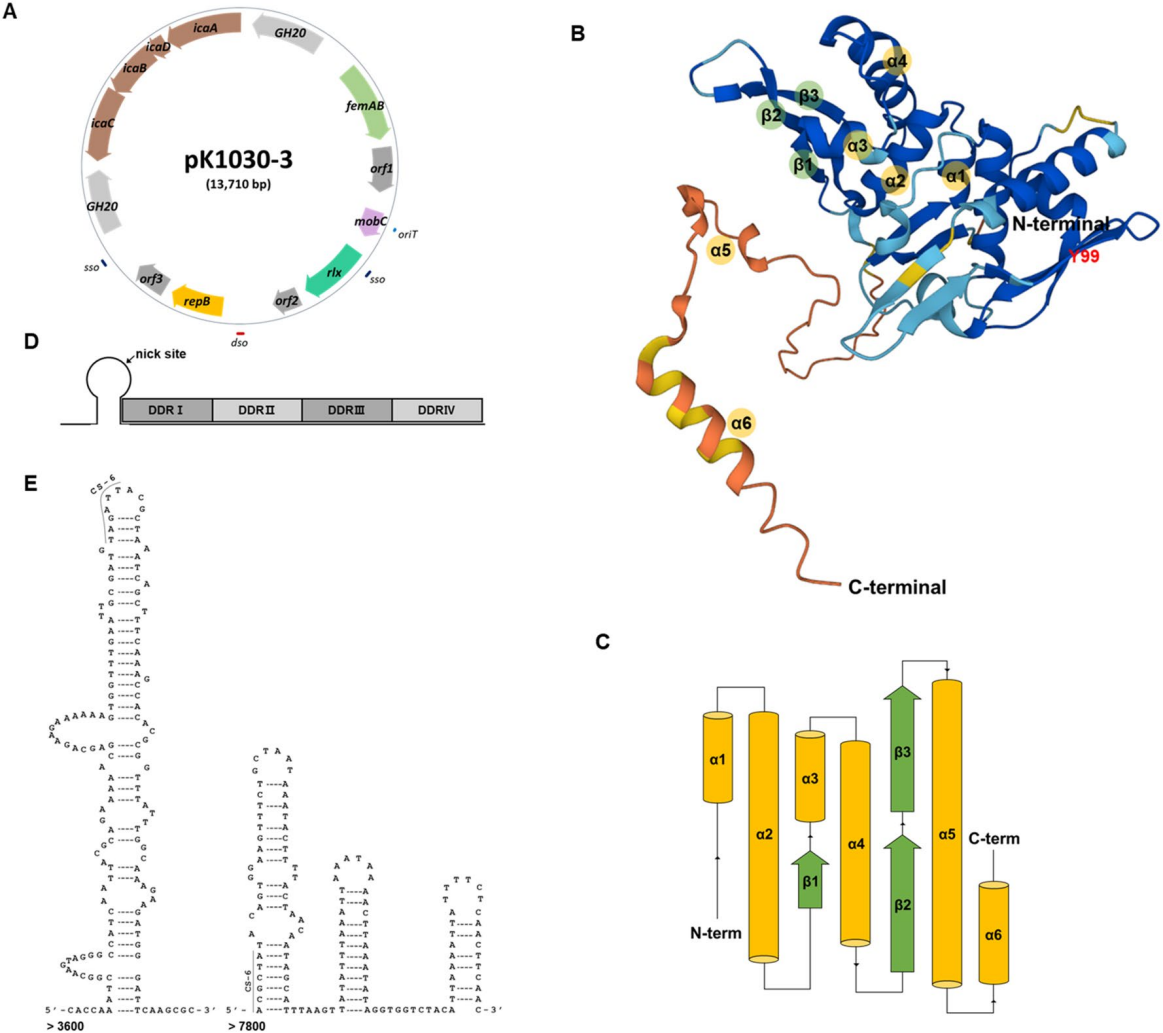
Circular representation of plasmid pKS1030-3 from *Staphylococcus equorum* strain KS1030 (**A**), three dimensional structure, (**B**) and domain architecture (**C**) of putative Rep, and gene structure of *dso* (**D**), and *sso* (**E**) in the plasmid. In (**A**), genes including open reading frames and genetic elements for replications are represented by the coloured arrows on the inner circle and bar on the outer circle, respectively. In (**B**), helix–turn–helix motif was presented by fold of α-helix and β-sheet. In (**D**), the nick site of *dso* was indicated by black arrows. In (**E**), inverted repeats and their stem-loops are displayed as arms and the CS-6 is shown using a line. Abbreviations: DDR, distal direct repeat.

*S. equorum* strain KS1030 harbours four plasmids: pKS1030-1, pKS1030-2, pKS1030-3, and pSELNU1^12^. pKS1030-3 (13,583 bp) contains 14 open reading frames (ORFs). Eleven of these ORFs were assigned putative functions via in silico sequence analysis (Fig. 1A and Table 1). The other three ORFs show high similarity to proteins of unknown function, or have no relatives in public databases (Table 1). The overall G + C content of the pKS1030-3 nucleotide sequence (29.1 mol%) is lower than the values for chromosomal DNAs and typical plasmids in *S. equorum* strains (30.9–34.3 mol%). Comparative sequence analysis identified three representative putative gene systems within the pKS1030-3 genome: the plasmid replication system, a biofilm formation system, and an HGT system.

**Table 1:** Putative open reading frames of pKS1030-3 and their functions.

Gene name	Gene locus	Position strand	Protein length (aa)	Product	Most relevant homology		
					Number of identities/number examined (%)	Organism	GenBank accession number
*GH20*	JL104_RS14620	−	344	Family 20 glycosylhydrolase	344/344 (100)	*Staphylococcus saprophyticus* SDC2	WP_165844925
	JL104_RS14625	+		tRNA-other			
*femAB*	JL104_RS14630	+	412	Aminoacyltransferase	411/412 (99)	*S. ureilyticus* NBRC 109,766	WP_046467876
*orf1*	JL104_RS14635	+	227	Hypothetical protein	226/227 (99)	*S. equorum* OffWhite_SAM	WP_069833254
*mobC*	JL104_RS14640	+	127	Plasmid mobilization relaxosome protein MobC	124/127 (98)	*S. casei* DSM 15,096	PNZ62186
*rlx*	JL104_RS14645	+	319	Relaxase/mobilization nuclease domain-containing protein	311/319 (97)	*S. equorum* SNUC 115	WP_119627660
*orf2*	JL104_RS14650	+	161	Hypothetical protein	152/161 (98)	*Staphylococcus* sp. GDY8P100P	WP_204171950
*repB*	JL104_RS14655	+	286	RepB family plasmid replication initiator protein	239/247 (97)	*S. equorum* 962_6	WP_069813166
*orf3*	JL104_RS14660	+	165	Hypothetical protein	158/169 (95)	*S. saprophyticus* GDH8C90P	WP_194379263
*GH20*	JL104_RS14665	+	306	Family 20 glycosylhydrolase	305/306 (99)	*S. cohnii* SE4.2	OIS27838
*icaC*	JL104_RS14670	−	350	Polysaccharide intercellular adhesin biosynthesis/export protein IcaC	350/350 (100)	*S. cohnii* SE4.3	WP_069820520
*icaB*	JL104_RS14675	−	284	Intercellular adhesin biosynthesis polysaccharide *N*-deacetylase	284/284 (100)	*Staphylococcus* sp. GDK8D68P	WP_069820518
*icaD*	JL104_RS14680	−	102	Intracellular adhesion protein IcaD	73/73 (100)	*S. saprophyticus* GDY8P136P	WP_194375771
*icaA*	JL104_RS14685	−	409	Poly-$\upbeta$-1,6 *N*-acetyl-D-glucosamine synthase	408/409 (99)	*S. arlettae* N283	WP_069820516

Position strand: +, forward; −, reverse.

### Replication system of pKS1030-3

The typical replication system of a plasmid via the rolling-circle mechanism in Gram-positive bacteria contains a replication protein-encoding gene (*repB*), a double-stranded origin of replication (*dso*), a single-stranded origin of replication (*sso*), and an origin of transfer (*oriT*)^26^. pKS1030-3 contains these genetic elements (Fig. 1).

The putative RepB protein encoded within pKS1030-3 comprises 286 amino acids and exhibits 96.5% and 95.1% sequence identity with the RepB family plasmid replication initiator proteins encoded by *S. equorum* 876_5 (WP_129651037) and *Companilactobacillus halodurans* TMW 1.2172 (WP_153386870), respectively. In silico analysis revealed that pKS1030-3 RepB contains a helix–turn–helix (HTH) motif in the central part of the protein, which is expected in replication proteins for rolling-circle replication^27^. The putative HTH motif resembles the winged HTH motif that binds with DNA (Fig. 1B). Additionally, pKS1030-3 RepB contains the conserved tyrosine residue (residues 99 in RepB) which commonly attacks the phosphodiester bond of the DNA for nicking (Fig. 1B)^28^. However, three conserved motifs (G, T, and H-U (hydrophobic amino acid)-H), which are commonly detected in replication proteins for rolling-circle replication, were not detected in the RepB of pKS1030-3^29,30^. A putative promoter, a − 35 region (5′-TTGCCA-3′, nucleotides 5619–5624) and a − 10 region (5′-TATTTA-3′, nucleotides 5644–5649) were detected upstream of *repB* in pKS1030-3, as was a Shine–Dalgarno sequence (5′-AGGAG-3′, nucleotides 5763–5767).

The Rep protein recognises the *dso*-containing nick site to generate single strand for rolling circle replication^31,32^. The *dso* sequence had a stem-loop structure containing the nick site that matches a consensus pattern (tACTAC gac-x-cccc-x(3)-GTg) and several copies of direct repeats^31,32^. The pKS1030-3 *dso* site (nucleotides 5417–5438) is located upstream of the start codon in the *repB* gene and contains the characteristic four distal direct repeat sequences, inverted repeat sequence, and nick site (Fig. 1D).

Additionally, pKS1030-3 possesses two *sso*s (nucleotides 3515–3657 and 7957–8080, respectively) (Fig. 1E), which are the initiation sites of lagging-strand synthesis and have a 6-bp consensus sequence (CS-6; 5′-TAGCGt/a-3′)^33^. Generally, *sso* sites contain extensive palindromic sequences that can form a folded structure^34,35^—we detected these palindromic sequences in pKS1030-3 *sso* when an RNA secondary structure prediction program (RNAstructure, v.6.4) was used (http://rna.urmc.rochester.edu).

### Biofilm formation system of pKS1030-3

Four *ica* genes (*icaA*, *icaB*, *icaC*, and *icaD*, constituting the *ica* operon) contribute to the production of exopolysaccharide (partially *N*-acetylated linear β-1–6-linked glycosaminoglycan), which is involved in biofilm formation by staphylococcal speceis^36^. The *ica* operon was detected in pKS1030-3 (nucleotides 9116–12579) and strain KS1030 forms biofilms (Fig. 1A). Comparative genomic analysis with two other *S. equorum* strains showed that the *ica* operon was present in strain KS1030, but absent from strains C2014 and KM1031(Supplementary Table 2).

**Figure 2:**
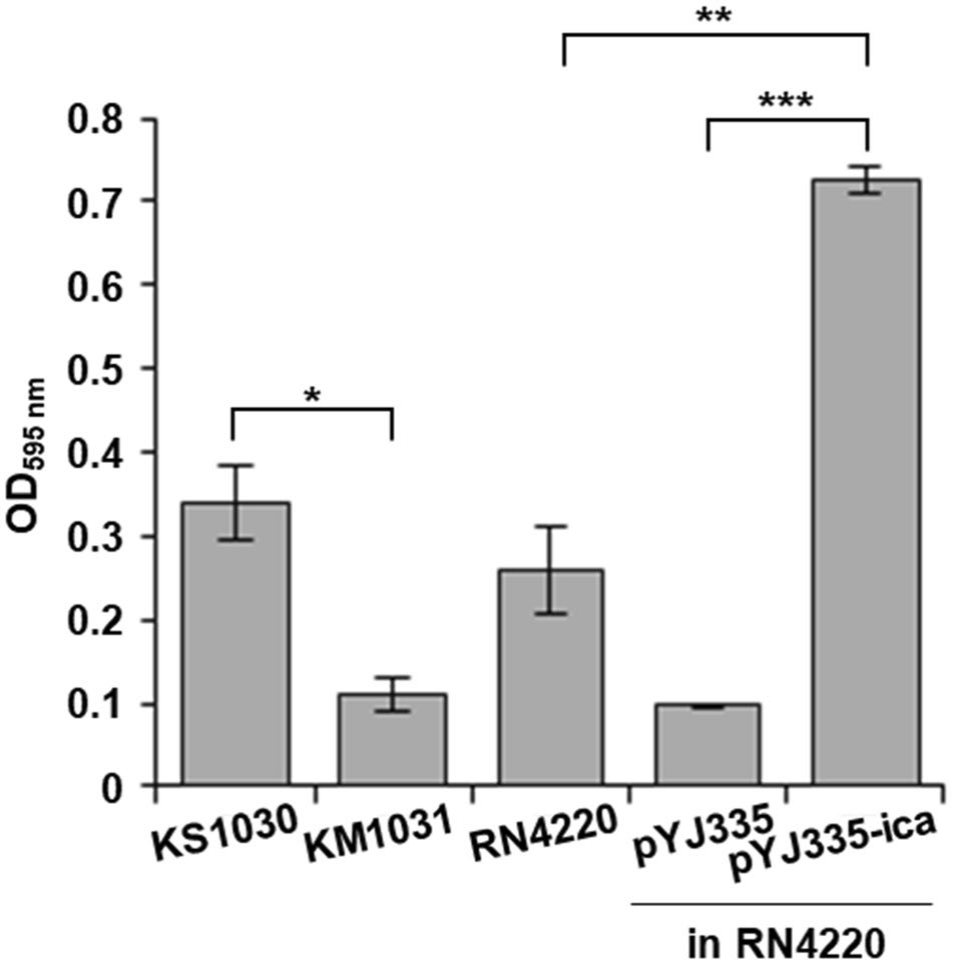
Effect of the *ica* operon from pKS1030-3 on biofilm formation. *S. equorum* strain KS1030 served as the positive control and donated the *ica* operon for construction of pYJ335-ica, which was expressed in *S. aureus* RN4220. *S. equorum* strain KM1031, which does not possesses the *ica* operon, served as a negative control. The means optical density values of the stained biofilms were calculated from three biological replicates run in duplicate. Statistical relevance was analysed using Duncan’s multiple range test; *, ** and ***indicate *p* < 0.05, *p* < 0.001 and *p* < 0.0001, respectively.

*S. equorum* strain KS1030 showed about three times higher biofilm formation capacity of strain KM1031 (Fig. 2). These results suggested that the *ica* operon of pKS1030-3 contributes to the biofilm ability of strain KS1030. Thus, the *ica* operon of pKS1030-3 was inserted into pYJ335 to construct pYJ335-ica, which was introduced into *S. aureus* RN4220. The biofilm formation ability of *S. aureus* RN4220 containing pYJ335-ica was confirmed to be about 2.8 and 7.6 times higher that of the control strain RN4220 and RN4220 containing pYJ335, respectively (Fig. 2). These results suggest that the *ica* genes in pKS1030-3 contribute to the biofilm formation ability of *S. equorum* strain KS1030.

In generally recognised as safe (GRAS) lactic acid bacteria, exopolysaccharides contribute to beneficial effects such as improvement of the texture of food, antitumour activity, and survival of probiotic strains in the gut^37^. However, in clinical isolates, exopolysaccharides contribute to biofilm formation, which is an important virulence factor via protection of bacteria from innate host defenses^36,38^. Biofilms contribute to the enhancement of AR and bacterial pathogenicity^36,39^. Genomic analysis of *S. equorum* KS1030 revealed that this strain does not possess virulence factors such as α-hemolysin, β-hemolysin, or enterotoxin genes, which have been detected in pathogenic *S. aureus*^40,41^. *ica* operon non-possessor strain KM1031 had a higher minimum inhibitory concentration of lincomycin than strain KS1030^10^, indicating that the *ica* operon did not contribute to the enhancement of lincomycin resistance in strain KS1030. Strain KS1030 is avirulent, so the *ica* operon by itself appears not to be an important virulence factor.

### HGT system of pKS1030-3

**Figure 3:**
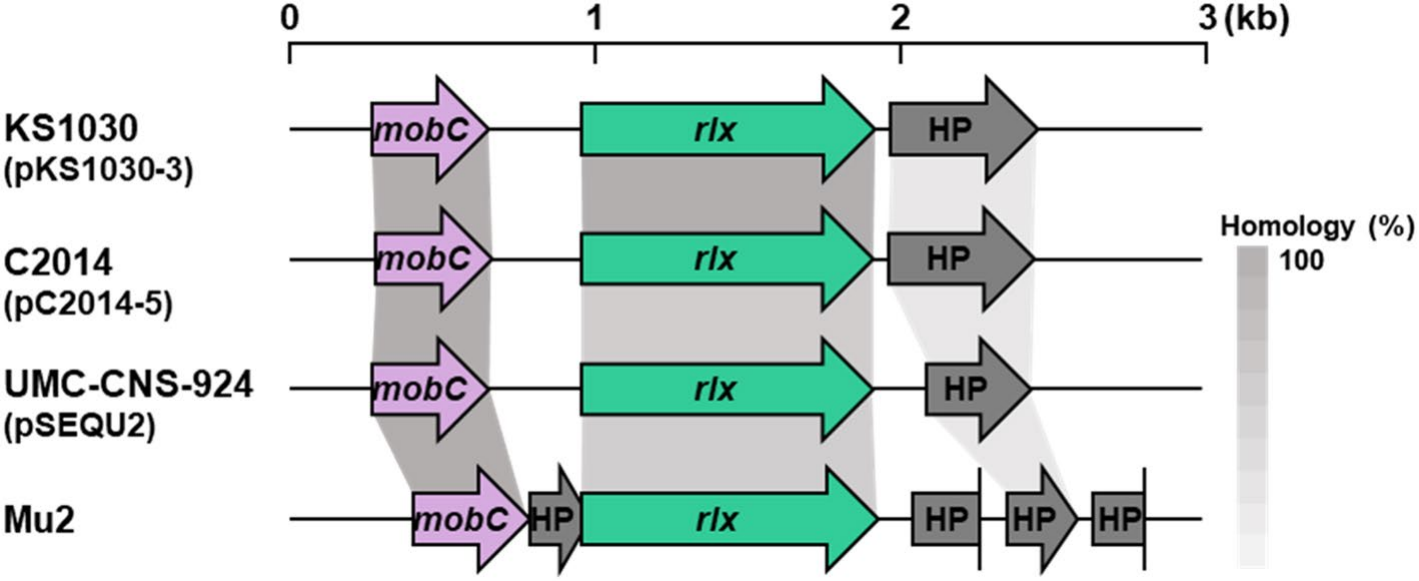
Relaxase (*rlx*) genes and their flanking regions found in different strains of *S. equorum*. The grey shading indicates the homology of deduced amino acid sequences between the genes. Abbreviations: *mobC*, mobilization protein gene; HP, hypothetical protein-coding gene.

HGT in Gram-positive bacteria can occur via conjugation. Commonly, DNA containing an *oriT* site, a relaxase (*rlx*), and a secretion system (such as the T4SS) is needed for conjugation^42^. The *rlx* gene of strain KS1030 is encoded on plasmid pKS1030-3. Comparative genomic analysis showed that the *rlx* gene in pKS1030-3 is a strain-specific gene, i.e., it was present in strains KS1030, C2014, UMC-CNS-924, and Mu2 but absent from strains KM1031 and KS1039 (Fig. 3). In addition, strain-specific putative mobilization protein genes, *mobC* and *orf2* (hypothetical protein), were located in flanking regions of the *rlx* gene (Fig. 1A). Interestingly, those genes were identified on the plasmid: pKS1030-3 in strain KS1030, pC2014-5 in strain C2014 and pSEQU2 in strain UMC-CNS-924, except strain Mu2. Genome of Mu2 is at the draft level, not at the complete genome, so it was not clear the position of those genes.

**Figure 4:**
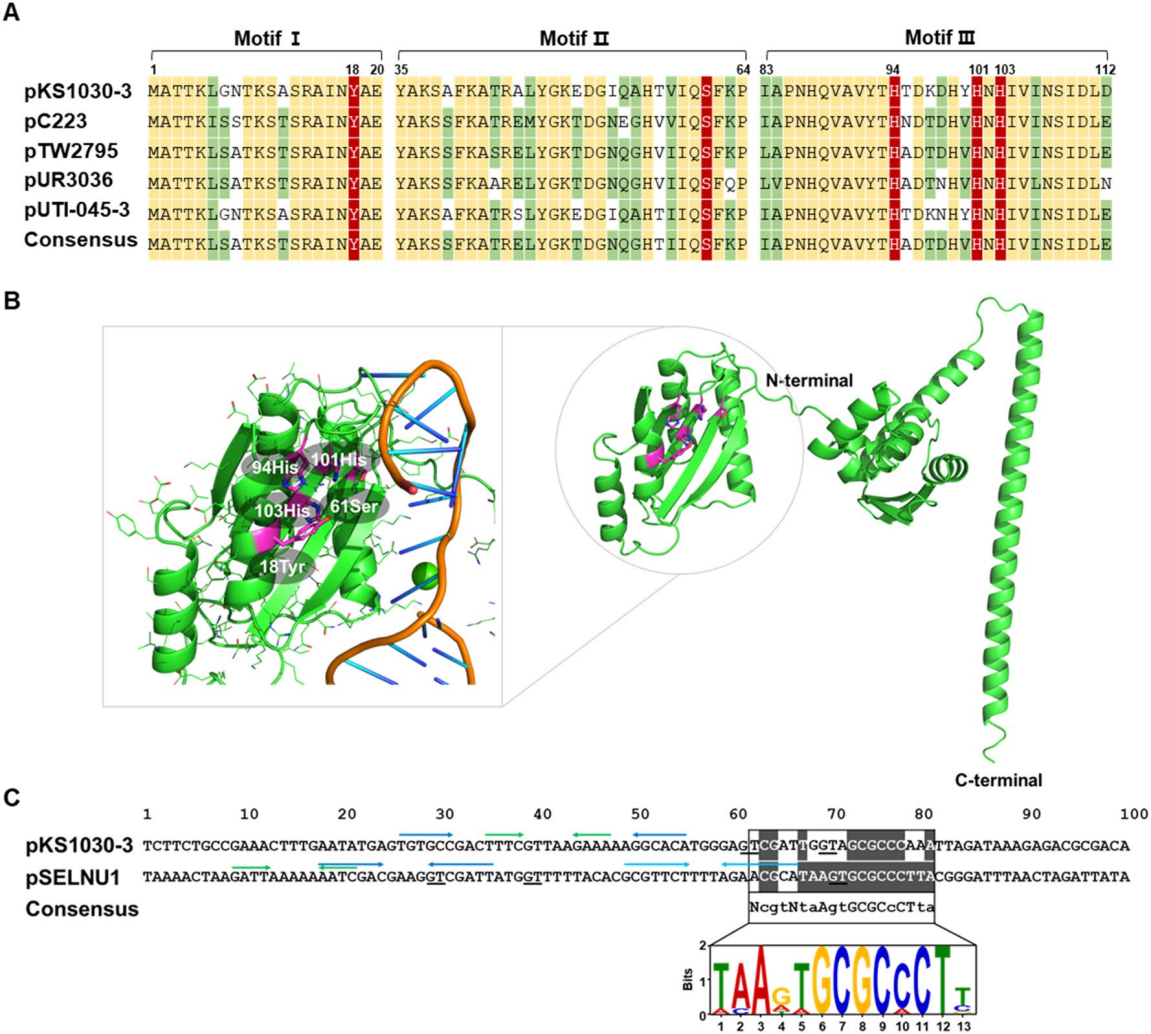
Relaxase and putative *oriT* sites of *S. equorum*. Alignment of sequence of *rlx* in pKS1030-3 with relevant regions of sequence from several plasmids (**A**), Three dimensional structure of putative Rlx (**B**), and putative *oriT* sequences of pKS1030-3 and pSELNU1 from *S. equorum* strain KS1030 (**C**). In (**A**), key conserved amino acids in the three conserved motifs of Rlx are indicated by white on red. Black on yellow: conserved residue; black on green: similar amino acid residues. In (**B**), key conserved amino acids are indicated by pink colour. In (**C**), inverted sequences are indicated by arrows of the same colour and the same as the consensus sequence is marked with white letters on a black background.

Relaxase is key enzyme of plasmid transfer initiation. Relaxase binds covalently to the recognition site of *oriT* and then cleaves the nick site of *oriT*; this complex is subsequently guided to the DNA transport machinery (such as the T4SS)^41^. The putative relaxase protein encoded by pKS1030-3 comprises 319 amino acids and exhibits 97.5% sequence identity with the relaxase (RIL47000.1) encoded on *S. equorum* SNUC 115 (Table 1). The deduced relaxase of pKS1030-3 has three conserved motifs that are identified in the MOB_p_ family among several mobilization families^43^. The relaxase in pKS1030-3 displayed the catalytic tyrosine residues (Y18) in motif I, a serine residue (S61) in motif II, and a histidine triad (H94, H101, and H103) in motif III (Fig. 4A). A conserved Y18 in motif I has been presumed to initiate the cleavage reaction via attack on the scissile DNA phosphodiester bond, and the conserved serine in motif II might be implicated in the interaction of the relaxase with the 3′-end of the nick DNA^44^. The histidine triad is involved in coordination a metallic cofactor such as a Mg^2+^ ion, essential in the cleavage mechanism^30^. The relaxase predicted using AlphaFold had a structure in which N-terminal and C-terminal were distinguished (Fig. 4B). And conserved motifs were located on the N-terminal, and it was able to predict the structure of binding with DNA (Fig. 4B). Relaxase recognises and binds to a specific DNA binding sequence, *oriT*, on the plasmid, while Rep recognises the *dso*. The *oriT* in Gram-positive bacteria has inverted sequence containing nick site^45^, and/or consensus sequence 5′-NcgtNtaAgtGCGCcCTta-3′^46^. Putative *oriT* sequence was detected in pKS1030-3 (Fig. 4C), these sequences was contained inverted sequences containing nick site, GT, and non-perfect consensus sequence.

Interestingly, a putative *oriT* site in pSELNU1 was more perfect matched with consensus sequences, even though this is not a conjugative plasmid (Fig. 4C). In addition, it was confirmed that it has three inverted sequences. These results suggest that the relaxase from pKS1030-3 might recognise the *oriT* sequence of pSELNU1, and then be trans-acting in the transfer of pSELNU1 into other bacteria. Although studies on the horizontal gene transfer of non-conjugation plasmids are not clear yet, results have been reported that they are transferred through trans-acting of relaxase from conjugal plasmids or sequences that mimic the sequence of bonding plasmids^47,48^. Accordingly, we assumed that the relaxase of pKS1030-3 might be involved the HGT of pSELNU1 via trans-acting and tried to prove this. A *mobC* gene was identified upstream of the *rlx* gene in plasmid pKS1030-3 (Fig. 1A). MobC helps to determine the specificity of *oriT* recognition by relaxase, enhances its nicking activity, stimulates the ATPase activity of the coupling protein, and acts as a transcriptional regulator for conjugative transfer operons^49^. MobC is homologous to prokaryotic transcription factors of the ribbon–helix–helix (RHH) superfamily, and putative MobCs have a RHH motif according to primary sequence analysis^50^. The putative MobC of pKS1030-3 showed the RHH structure (Supplementary Fig. 1). Consequently, genomic analysis suggests that the *rlx*, *mobC*, and *oriT* of pKS1030-3 might contribute to HGT. Therefore, an approximately 2.3-kb fragment of pKS1030-3 containing *mobC*, *rlx*, and ORF2 was amplified and cloned into the staphylococcal shuttle vector pYJ335. ORF2 was included because it showed 93% similarity with the mobilization protein (WP_012817945) of *Staphylococcus* sp. 693.2 (although its highest similarity was with a hypothetical protein) (Table 1). The resulting plasmid was named pYJ335-rlx. pSELNU1, containing the *lnuA* gene, was used to confirm the horizontal transfer via trans-acting mobilization. Plasmids pYJ335-rlx and pSELNU1 were co-introduced into *S. aureus* RN4220, which does not normally possesses the relaxosome and is sensitive to lincomycin. *S. saprophyticus* KM1053, which is resistant to tetracycline, was used as the recipient strain^11^. Transconjugants via *S. aureus* RN4220 (pSELNU1 and pYJ335-rlx) showing lincomycin and tetracycline resistance were detected at a frequency of 3.5 × 10^−6^, while transconjugants via *S. aureus* RN4220 (pSELNU1) were detected at a frequency of just 8.3 × 10^−9^ (Table 2). No transconjugants via *S. aureus* RN4220 (pYJ335-rlx) were detected. These results indicate that the relaxase encoded by pKS1030-3 increased the horizontal transfer of pSELNU1 in a trans-acting manner. Nevertheless, there are still some issues to be resolved, such as the need for experimental proof of the binding of the relaxase with the *oriT* site, determination of the role(s) of MobC and ORF2 in the process, identification of the inducer for horizontal transfer, and identification of the secretion system for pSELNU1.

**Table 2:** In vitro transfer of pSELNU1 from *S. aureus* RN4220 to *S. saprophyticus* KM1053.

Plasmids in		Cell count (CFU/ml)			Transfer rate (T/R)
Donor strain	Recipient strain	Donor	Recipient (R)	Transconjugants (T)	
pSELNU1	−	9.4 × 10^8^	6.0 × 10^8^	5.0	8.3 × 10^−9^
pYJ335-rlx	−	5.0 × 10^8^	5.0 × 10^8^	ND	
pSELNU1 + pYJ335-rlx	−	6.4 × 10^8^	3.4 × 10^8^	1.2 × 10^3^	3.5 × 10^−6^

Donor strains were derived from *Staphylococcus aureus* RN4220 by electroporation of indicated plasmids. The recipient strain was *S. saprophyticus* KM1053. Cell counts were repeated three times independently and the mean value of the replicates is presented. − indicates that the strain did not possess a plasmid. ND, not detected; R, recipient; T, transconjugant; CFU, colony-forming units.

**Figure 5:**
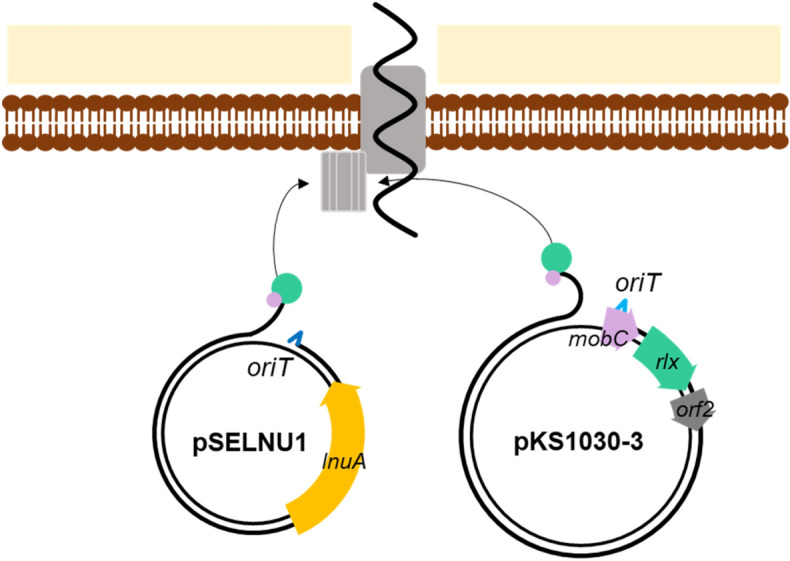
Working model for horizontal transfer of pSELNU1 by *S. equorum* strain KS1030, using the relaxase encoded in pKS1030-3 acting in trans. pKS1030-3 carries an origin of transfer (*oriT*; blue line) and encodes a DNA relaxase (*rlx*; green box) as well as a coupling protein (*mobC*; pink box) and products for formation of the mating pore. Rlx and MobC were indicated by green and pink circle, respectively. HP and secretion systems were grayed out because their genes or functions were not inferred. Brown was the cell membrane, beige meant peptidoglycan. pSELNU1 also carries an *oriT* that is recognised by the relaxase of pKS1030-3 and forms the mating pore.

In conclusion, *S. equorum* KS1030 shows strain-specific lincomycin resistance and biofilm formation properties^9^. Previously, we determined that the lincomycin resistance derives from acquired plasmid pSELNU1, which encodes the lincomycin resistance gene *lunA* without mobile elements such as relaxase, from *S. equorum* KS1030^10^. In the current study, we identified that the relaxase and *ica* operon of plasmid pKS1030-3 and those genes involved the HGT of pSELNU1 and biofilm formation by *S. equorum* KS1030, respectively. Notably, in the transfer, the HGT elements encoded in pKS1030-3 are trans-acting (Fig. 5). Recently, there have been many concerns that strains exhibiting acquired antibiotic resistance are unsafe^1,51,52^. However, current results showed that even if strain has acquired antibiotic resistance gene, it cannot be transfer horizontally into other species without a mobile element such as relaxase. These results were also confirmed in other experimental results. *S. equorum* strain KM1031 has plasmid pSELNU3 encoding the *lnuA* gene that is resistant to lincomycin^10^. However, *S. equorum* strain KM1031 did not encoded the relaxase gene and did not showed the horizontal gene transfer of pSELNU3 into other strains and/or species. These results can be assumed that horizontal gene transfer did not occurred without mobile elements even if the acquired antibiotic resistance gene is present. Therefore, to select the safe starter candidates in horizontal gene transfer of antibiotic resistance, it is necessary to check whether you have mobile elements at the same time as checking whether you have acquired antibiotic resistance genes. Fermented foods act as reservoirs and vehicles for large populations of living bacteria and have been proposed as possible sources of antibiotic-resistant bacteria^53–55^. It is not yet known if pSELNU1 could transfer between *S. equorum* strains, or into other species, if *S. equorum* KS1030 containing pSELNU1 were used as a starter strain. However, if such horizontal plasmid transfer can occur during fermentation, there is a need to control it.

## Supplementary Information

Supplementary Information.

## Data Availability

The genome sequences used during the current study are available in in NCBI data base (http://ncbi.nlm.nih.gov/genomes): CP068576–CP068580 for *S. equorum* strain KS1030; CP013980–CP013983 for strain KM1031; CP013714–CP013719 for strain C2014; CP013114 for strain KS1039; CAJL01000001–CAJL01000030 for strain Mu2; and AVBD01000001–AVBD01000039 for strain UMC-CNS-924. All relevant data are within the manuscript and its supporting information files.
