# Supplementary material for: Staphylococcus equorum plasmid pKS1030-3 encodes auxiliary biofilm formation and trans-acting gene mobilization systems
Source: Sci Rep. 2023 Jul 10;13:11108. doi: 10.1038/s41598-023-38274-8 (PMC10333310; doi:10.1038/s41598-023-38274-8)
Supplement: Supplementary file 1 — Supplementary Information. [file 41598_2023_38274_MOESM1_ESM.pdf]

Supplementary Fig. 1. Predicted gene structure of *mobC* in pKS1030-3. Three dimensional structure of putative *mobC* was obtained by the AlphaFold prediction program.

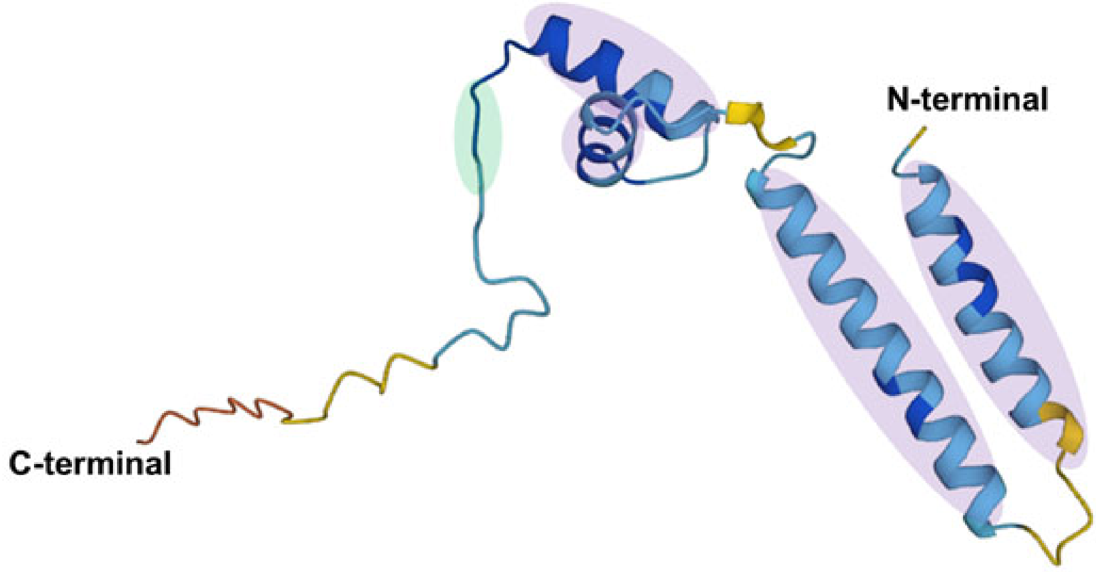

Supplementary Table 1. Oligonucleotide primer sequences for cloning of biofilm formation and relaxase genes.

Primer	Sequence (5'→3')	Expected size (bp)
ica-F	CCG CTC GAG CGA GCA TGC TAC AGG CAT GAG	3,760
ica-R	CCG CTC GAG CCA TAT CGA TAT GGA ATG CGC	
Rlx-F	CCG ATA TCC TCA TCT CCA TCT TAT GGC	2,360
Rlx-R	CCG CTC GAG CAA ACT ATG AGT GGC TAG	

Underlines indicate nucleotide participating in restriction sites

Supplementary Table 2. List of comparative genomes for genes located in plasmids possessed by *S. equorum* strain KS1030 with strains C2014 and KM1031. These list were obtained by the Efficient Database framework for comparative Genome Analyses using BLST score Rations (EDGAR). Each two genes were considered orthologous when revealing a bidirectional best BLAST hit with a single score ratio value threshold of at least 32% for orthology estimation.

Product	KS1030	C2014	KM1031
Recombinase family protein	JL104_RS14225[S1]		
Multicopper oxidase domain-containing protein	JL104_RS14230[S1]		
Hypothetical protein	JL104_RS14235[S1]		
Cation transporter	JL104_RS14240[S1]		
Cadmium-translocating P-type ATPase	JL104_RS14245[S1]		
DNA starvation/stationary phase protection protein	JL104_RS14250[S1]		
Cation transporter	JL104_RS14255[S1]		
Recombinase family protein	JL104_RS14260[S1]		
DDE-type integrase/transposase/recombinase	JL104_RS14265[S1]		
AAA family ATPase	JL104_RS14270[S1]		
CadD family cadmium resistance transporter	JL104_RS14275[S1]		AWC34_RS12890[M1]
Cadmium-translocating P-type ATPase CadA	JL104_RS14280[S1]		
Cd(II)/Pb(II)/Zn(II)-sensing metalloregulatory transcriptional repressor CadC	JL104_RS14285[S1]		
Recombinase family protein	JL104_RS14290[S1]	AVJ22_RS03770	AWC34_RS12960[M1]
Arsenate reductase (thioredoxin)	JL104_RS14295[S1]		AWC34_RS12955[M1]
Arsenite efflux transporter membrane subunit ArsB	JL104_RS14300[S1]		AWC34_RS12950[M1]
Metalloregulator ArsR/SmtB family transcription factor	JL104_RS14305[S1]		AWC34_RS12945[M1]
FAD-dependent oxidoreductase	JL104_RS14310[S1]		AWC34_RS12940[M1]
Arsenical pump-driving ATPase	JL104_RS14315[S1]		AWC34_RS12935[M1]
Arsenite efflux transporter metallochaperone ArsD	JL104_RS14320[S1]		AWC34_RS12930[M1]
Hypothetical protein	JL104_RS14325[S1]		AWC34_RS12925[M1]
Metalloregulator ArsR/SmtB family transcription factor	JL104_RS14330[S1]	AVJ22_RS03795	AWC34_RS12920[M1]
Permease	JL104_RS14335[S1]		AWC34_RS12915[M1]
Hypothetical protein	JL104_RS14340[S1]		
DUF536 domain-containing protein	JL104_RS14345[S1]		AWC34_RS12905[M1]
Replication initiator protein A	JL104_RS14350[S1]	AVJ22_RS14035[C3]	AWC34_RS12900[M1]
Hypothetical protein	JL104_RS14360[S1]	AVJ22_RS14680[C3]	
GNAT family N-acetyltransferase	JL104_RS14365[S1]		
HTH domain-containing protein	JL104_RS14370[S1]		AWC34_RS12880[M1]
Hypothetical protein	JL104_RS14375[S1]		
Type I toxin-antitoxin system Fst family toxin	JL104_RS14380[S1]	AVJ22_RS14350	AWC34_RS13615
2,3-butanediol dehydrogenase	JL104_RS14385[S1]		AWC34_RS13225[M2]
Hypothetical protein	JL104_RS14390[S1]		
Acyl-CoA thioesterase	JL104_RS14395[S1]		
LysR family transcriptional regulator	JL104_RS14400[S1]		

Product	KS1030	C2014	KM1031
Heavy metal translocating P-type ATPase	JL104_RS14410[S1]		AWC34_RS13000[M1]
Cation transporter	JL104_RS14415[S1]		AWC34_RS12995[M1]
DNA starvation/stationary phase protection protein	JL104_RS14420[S1]		AWC34_RS12990[M1]
Crp/Fnr family transcriptional regulator	JL104_RS14425[S1]		AWC34_RS12985[M1]
Transposase	JL104_RS14430[S1]		AWC34_RS13450[M1]
Transposase	JL104_RS14435[S1]		AWC34_RS12980[M1]
Helix-turn-helix domain-containing protein	JL104_RS14440[S1]		
Sulfite exporter TauE/SafE family protein	JL104_RS14445[S1]		AWC34_RS12825[M1]
Persulfide-sensing transcriptional repressor CstR	JL104_RS14450[S1]		AWC34_RS13010[M1]
Persulfide response sulfurtransferase CstA	JL104_RS14455[S1]		AWC34_RS13015[M1]
Persulfide dioxygenase-sulfurtransferase CstB	JL104_RS14460[S1]		AWC34_RS12840[M1]
Protein deglycase HchA	JL104_RS14465[S2]		
NAD(P)H-quinone oxidoreductase	JL104_RS14470[S2]		
MerR family transcriptional regulator	JL104_RS14475[S2]		
Thioredoxin family protein	JL104_RS14480[S2]		
Thioredoxin	JL104_RS14485[S2]		
OsmC family protein	JL104_RS14490[S2]		
12-oxophytodienoate reductase	JL104_RS14495[S2]		
Carboxymuconolactone decarboxylase family protein	JL104_RS14500[S2]		
Thioredoxin-disulfide reductase	JL104_RS14505[S2]		
TetR/AcrR family transcriptional regulator	JL104_RS14510[S2]		
Type I toxin-antitoxin system Fst family toxin	JL104_RS14545[S2]		
Replication initiator protein A	JL104_RS14570[S2]	AVJ22_RS13890[C2]	
DUF536 domain-containing protein	JL104_RS14575[S2]	AVJ22_RS13885[C2]	
CueP family metal-binding protein	JL104_RS14585[S2]		
Copper-translocating P-type ATPase	JL104_RS14590[S2]		
APC family permease	JL104_RS14595[S2]		
YdhK family protein	JL104_RS14600[S2]		
Four-helix bundle copper-binding protein	JL104_RS14605[S2]	AVJ22_RS13320[C1]	
Multicopper oxidase domain-containing protein	JL104_RS14610[S2]		
Family 20 glycosylhydrolase	JL104_RS14620[S3]		
Aminoacyltransferase	JL104_RS14630[S3]		
Hypothetical protein	JL104_RS14635[S3]		
Plasmid mobilization relaxosome protein MobC	JL104_RS14640[S3]	AVJ22_RS14090[C5]	
Relaxase/mobilization nuclease domain-containing protein	JL104_RS14645[S3]	AVJ22_RS14085[C5]	
Hypothetical protein	JL104_RS14650[S3]	AVJ22_RS14080[C5]	
RepB family plasmid replication initiator protein	JL104_RS14655[S3]	AVJ22_RS14075[C4]	
Replication-associated family protein	JL104_RS14660[S3]		
Family 20 glycosylhydrolase	JL104_RS14665[S3]		
Polysaccharide intercellular adhesin biosynthesis/export protein IcaC	JL104_RS14670[S3]	AVJ22_RS01555	
Intercellular adhesin biosynthesis polysaccharide N-deacetylase	JL104_RS14675[S3]		
Intracellular adhesion protein D	JL104_RS14680[S3]		
Poly-beta-1,6 N-acetyl-D-glucosamine synthase	JL104_RS14685[S3]		
Protein rep	JL104_RS14690[S4]	AVJ22_RS14250[C3]	AWC34_RS13295[M3]

Product	KS1030	C2014	KM1031
Lincosamide nucleotidyltransferase Lnu(A)	JL104_RS14695[S4]		AWC34_RS13300[M3]

The indication of the superscript means the plasmid in which the gene is located. The unmarked one exists in the chromosome. [S1], pKS1030-1; [S2], pKS1030-2; [S3], pKS1030-3; [S4], pSELNU1; [C1], pC2014-1; [C2], pC2014-2; [C3], pC2014-3; [C4], pC2014-4; [C5], pC2014-5; [M1], KM1031 plasmid 1; [M2], KM1031 plasmid 2; [M3], KM1031 plasmid 3 (pSELNU3).
